# Carboxypeptidase A6 was identified and validated as a novel potential biomarker for predicting the occurrence of active ulcerative colitis

**DOI:** 10.1111/jcmm.15517

**Published:** 2020-06-22

**Authors:** Haizhou Wang, Meng Zhang, Mengna Zhang, Fan Wang, Jing Liu, Qiu Zhao

**Affiliations:** ^1^ Department of Gastroenterology Zhongnan Hospital of Wuhan University Wuhan China; ^2^ Hubei Clinical Center and Key Lab of Intestinal and Colorectal Diseases Wuhan China

**Keywords:** active UC, anti‐TNF treatment, CPA6, WGCNA

## Abstract

Ulcerative colitis (UC) is a chronic, highly heterogeneous intestinal inflammation with changes in epithelial function and tissue damage. However, the pathogenesis is still unclear between active UC and inactive UC. Herein, weighted gene co‐expression network analysis was applied to explore the gene modules related to active UC. Gene set enrichment analysis (GSEA) and gene set variation analysis (GSVA) were used to further investigate the underlying mechanism of selected genes. We found that in the blue module (*r* = −.72), carboxypeptidase A6 (CPA6) was chosen to validate because of its high intra‐modular connectivity and module membership. In the test sets, the expression level of CPA6 was down‐regulated in active UC compared with inactive UC and normal colon. Furthermore, CPA6 expression was decreased primarily in the descending colon and only in mucosa affected by active UC. The receiver operating characteristic curve indicated that CPA6 expression had a performed well in diagnosing active UC from inactive UC (area under the curve = 0.99). Importantly, anti‐tumour necrosis factor (TNF) treatment (infliximab and golimumab) significantly increased the CPA6 expression. Finally, GSEA and GSVA found that extracellular matrix receptor, inflammatory response and epithelial‐mesenchymal transition were highly enriched in active UC with low CPA6 expression. In conclusion, CPA6 was identified and validated as a novel potential biomarker for predicting the occurrence of active UC, probably through regulating extracellular matrix or immune response.

## 1 INTRODUCTION

Ulcerative colitis (UC) is a complex, chronic intestinal inflammation accompanied by epithelial function and tissue damage.^1^ Currently, anti‐tumour necrosis factor (TNF) therapy is the first effective biological therapy and has been the first‐line treatment for patients with moderate to severe UC for 15 years.^2^ However, based on the epidemiological report in 2018, the incidence in many countries has exceeded 0.3% and is still increasing.^1^ This situation severely affects the quality of life of UC patients and places a tremendous burden on the national healthcare systems.^3^ Moreover, there are too many factors to induce the occurrence of active UC, such as immune response and inflammation,^4^ environmental factors,^5^ intestinal flora^6^ and intestinal barrier.^7^ Therefore, the exact mechanism of active UC is still complicated and further research is urgently needed.

In recent years, high‐throughput technology had been used to explore the relationship between differentially expressed genes (DEGs) and UC. As reported by Feng et al in 2017, a total of 233 DEGs were screened between UC patients and normal control by integrating 12 data sets. These genes were closely related to inflammation, immunity and fibrosis.^8^ Another study indicated 150 significantly DEGs were likely to involve in the UC development.^9^ These data showed the potential roles of DEGs in regulating UC. However, they only focused on the DEG screening and neglected the interconnection of genes with similar biological functions. Thus, more and more studies applied weighted gene co‐expression network (WGCNA), a biologically based algorithm to explore the key modules related to clinical traits.^10^, ^11^ Furthermore, the construction of gene co‐expression network was represented by nodes and edges to show the relationships between different genes. In this approach, highly connected genes were called as hub genes in a module, which were expected to be highly functional significant.^12^ For instance, Lin et al^13^ indicated that interleukin‐8 (IL‐8) and matrix metalloproteinase‐9 (MMP‐9) played crucial roles for the progression of UC. However, these studies only compared the relationship between normal mucosa and UC or active UC and did not the difference between inactive UC and active UC. Therefore, we firstly reanalysed the expression profile between active and inactive UC and constructed a co‐expression network using WGCNA algorithm to explore the hub genes related to active UC.

## 2 METHODS

### 2.1 Active UC data studies

**FIGURE 1:**
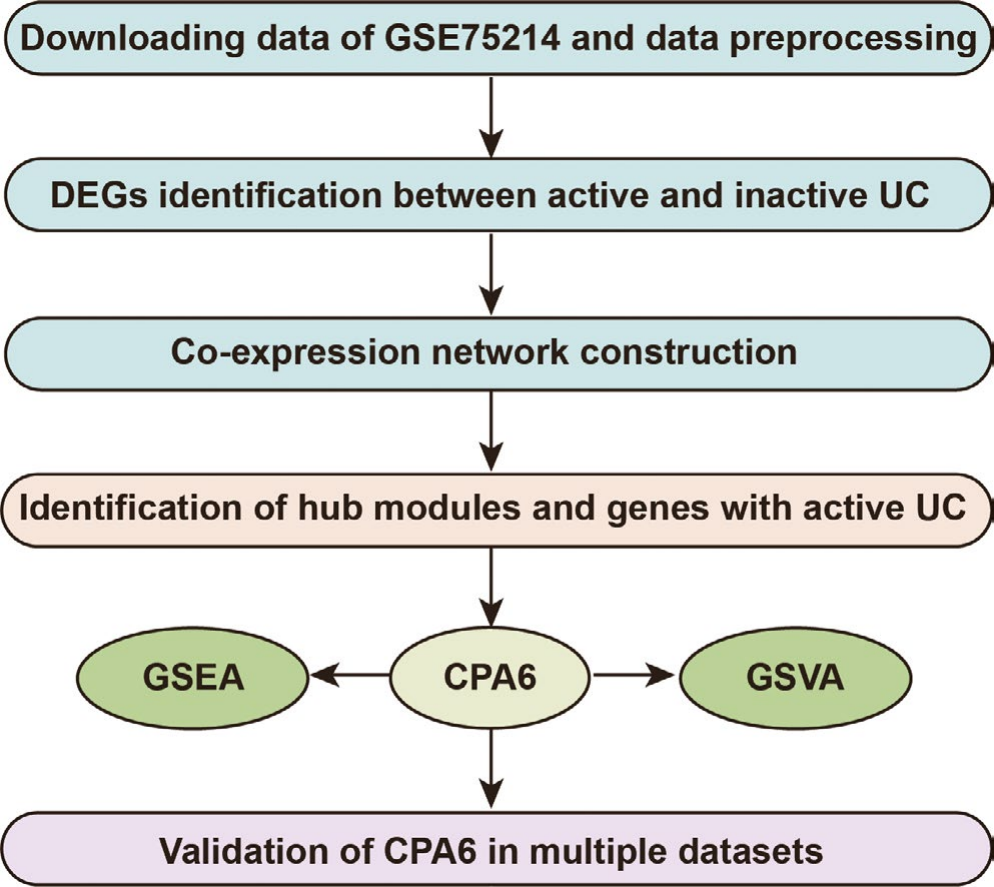
Flow chart of the whole procedures

The flow chart in this study was showed in Figure 1. The Gene Expression Omnibus (GEO) database (http://www.ncbi.nlm.nih.gov/geo/) was used to download the microarray data from UC. First, the GSE75214 data set, which contained 74 active UC and 23 inactive UC colon mucosa samples, was selected as a training set to construct the co‐expression network.^14^ Then, we selected several data sets as test sets to validate our results based on the following criteria: (a) active human UC samples; (b) data set type: expression profiling by array; (c) CPA6 expression profile data were contained; (d) total sample size >20. In brief, the GSE48958 data set included 13 UC (seven active and six inactive) patients and eight normal samples.^15^ The GSE94648 data set included peripheral whole blood from UC patients (17 active and eight inactive), as well as 22 non‐inflammatory controls.^16^ The GSE13367 data set contained eight active UC, nine inactive UC and 10 normal samples.^17^ A total of 43 mucosal biopsies were contained in GSE38713 data set, including 13 healthy controls, eight inactive UC, seven non‐involved active UC and 15 involved active UC.^18^ The GSE11223 data set collected colon biopsies from multiple sites in UC patients, including 32 ascending colons (11 with inflammation, 21 without inflammation), 34 descending colons (19 with inflammation, 15 without inflammation) and 58 sigmoid colons (33 with inflammation, 25 without inflammation).^19^ The GSE36807 data set included seven controls, 13 CD and 14 UC patients.^20^ The GSE87473 data set collected mucosal biopsies from paediatric (13 with severely UC, six with moderately UC) or adult patients (27 with severely UC, 60 with moderately UC).^21^

In addition, we searched several data sets with UC patients treated with anti‐TNF. The GSE23597 included 113 biopsy active UC samples treated with infliximab (IFX) or placebo at weeks 0, 8 and 30.^22^ The GSE16879 data set had a total of 48 UC patients and divided into 24 samples of IFX pretreatment and 24 samples after IFX treatment. And these samples were consisted of eight IFX responding and 16 IFX non‐responding samples.^23^ The GSE92415 data set included 183 UC patients treated with golimumab (GLM) and placebo for 6 weeks, which also be divided into GLM responding and non‐responding samples.

Moreover, UC‐related colon cancer data sets were also collected. The GSE4183 data set contained colonic biopsies of 15 patients with CRC, 15 with adenoma, 15 with IBD and eight healthy normal controls.^24^ The GSE3629 data set contained 53 UC patients, 10 had UC‐associated neoplastic lesions (UC‐Ca) and 43 patients had no neoplastic lesions (UC‐NonCa).^25^ The data set GSE37283 was comprised of five normal controls, four inactive UC and 11 UC with neoplasia.^26^ The mRNA expression data of colon adenocarcinoma (COAD) were also downloaded from The Cancer Genome Atlas (TCGA) database (https://genome-cancer.ucsc.edu/).

### 2.2 Data processing

The ‘Oligo’ R package was used to preprocess the raw data with RMA background correction, log2 transformation and quantile normalization.^27^ The ‘hugene10sttranscriptcluster.db’ R package was used to annotate all the probes. Additionally, GSE75214 was assessed by sample clustering according to the distance between different samples in Pearson's correlation matrices (Figure S1). No samples were removed under the cut‐off height of 40. For all test sets, after annotating the probes based on the annotation file of the corresponding platform, the expression of candidate hub genes was extracted from the matrix files.

### 2.3 DEG screening

The ‘limma’ R package was used to screen DEGs between active UC and inactive UC patients.^28^ A false discovery rate (FDR) of <0.05 and |log2 fold change (FC)| > 0.585 were selected as the cut‐off.

### 2.4 WGCNA construction

**FIGURE 2:**
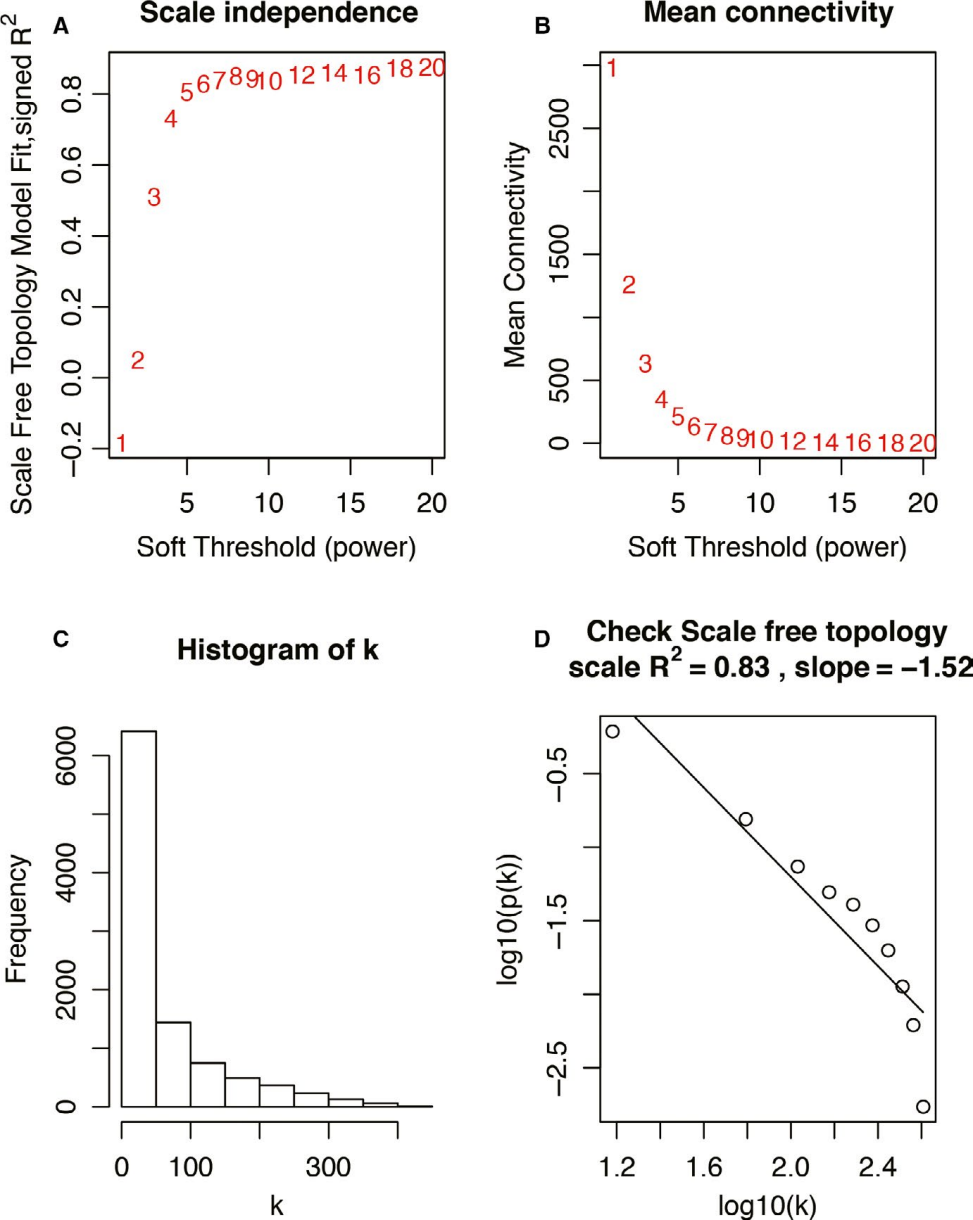
Determination of soft‐thresholding power in the co‐expression network. A, The scale‐free fit index was analysed for various soft‐thresholding powers (*β*). B, Analysis of the mean connectivity for various soft‐thresholding powers. C, Histogram of connectivity distribution when *β* = 8. D, Checking the scale‐free topology when *β* = 8

The ‘WGCNA’ R package was used to construct a co‐expression network based on the DEGs screened above.^29^ First, the Pearson's correlation matrices were calculated for all paired genes and a weighted adjacency matrix was constructed through a power function *a*_mn_ = **|** *c*_mn_ **|** *^β^* (*c*_mn_ = Pearson's correlation between gene m and gene n; *a*_mn_ = adjacency between gene m and gene n). Then, the soft threshold, parameter *β* was calculated, which emphasized strong correlations between genes and penalize weak correlations. In this study, a value of *β* = 8 (scale *R* ^2^ = .83) was applied to build a scale‐free network (Figure 2). Next, the adjacency was transformed into a topological overlap matrix (TOM) that can measure the network connectivity of genes and was defined as the sum of its adjacency with all other genes for network generation.^30^ Finally, average linkage hierarchical clustering was used to classify all DEGs with similar expression profiles into different modules, according to the TOM‐based dissimilarity measure with a minimum gene group size of 30 for the genes dendrogram.^31^

### 2.5 Key modules identification and hub genes

Herein, we used two methods to identify key modules associated with active UC. On one hand, gene significance (GS) was calculated by the log10 transformation of the *P*‐value in the linear regression analysis between active UC and expression profiles. The module significance (MS) was defined as the average GS for all the genes in the key modules. On the other hand, module eigengenes (MEs) were also considered as the first principal component in the principal component analysis for each module, and the expression patterns of all genes were summarized into a single characteristic expression profile within a given module. Finally, the key module (the blue module) was identified by both the highest absolute value of MS and the correlation between active UC and MEs. Meanwhile, to select hub genes in the key module, the genes in the blue module both with the higher absolute value of the Pearson's correlation (cor.standard > **|**0.85**|**) and module membership (MM) (cor.weighted > **|**0.85**|**) were considered as hub genes.^11^, ^29^, ^32^

### 2.6 Hub genes validation

The GEO data sets mentioned above were chosen to validate the relationship between hub genes and active UC. The ‘pROC’ R package was used to plot the receiver operating characteristic curve (ROC) curve and if the area under the ROC curve (AUC) >0.7, hub genes were defined to be able to distinguish active UC from inactive UC. The TCGA and Gene Expression Profiling Interactive Analysis (GEPIA2) (http://gepia2.cancer-pku.cn) databases were select to verify the correlation of hub genes with UC‐associated neoplastic lesions and colorectal cancer.

### 2.7 Gene set enrichment analysis (GSEA) and gene set variation analysis (GSVA)

To further explore the underlying mechanisms of CPA6, 74 samples of active UC in the GSE75214 were divided into low‐ and high‐expression groups based on the CPA6 expression. Then, GSEA was used to enrich KEGG pathways between the two groups. The NOM *P* value < .05 was used as the cut‐off threshold. In addition, GSVA was also applied to identified significant gene sets between low‐ and high‐CPA6 expression group. GSVA is a non‐parametric unsupervised method to assess the gene set enrichment using high‐throughput data. The ‘GSVA’ R package was used to transform the data from a gene by sample matrix to a gene set by sample matrix, and then calculated the enrichment scores each sample.^33^ The H.all.v6.2 was also used as the reference set. Comparison of low‐ and high‐expression group was performed using ‘limma’ package. Significant enriched gene sets were defined under the threshold of false discovery rate (FDR) <0.01 and mean score difference >0.2.

## 3 RESULTS

### 3.1 DEG screening

After data preprocessing and probe annotations, we obtained the expression matrices of GSE75214 with 18 821 genes and 97 samples. Finally, a total of 9911 DEGs (4139 up‐regulated and 5772 down‐regulated) were screened for network construction.

### 3.2 Construction of weighted gene co‐expression network and identification of key modules

**FIGURE 3:**
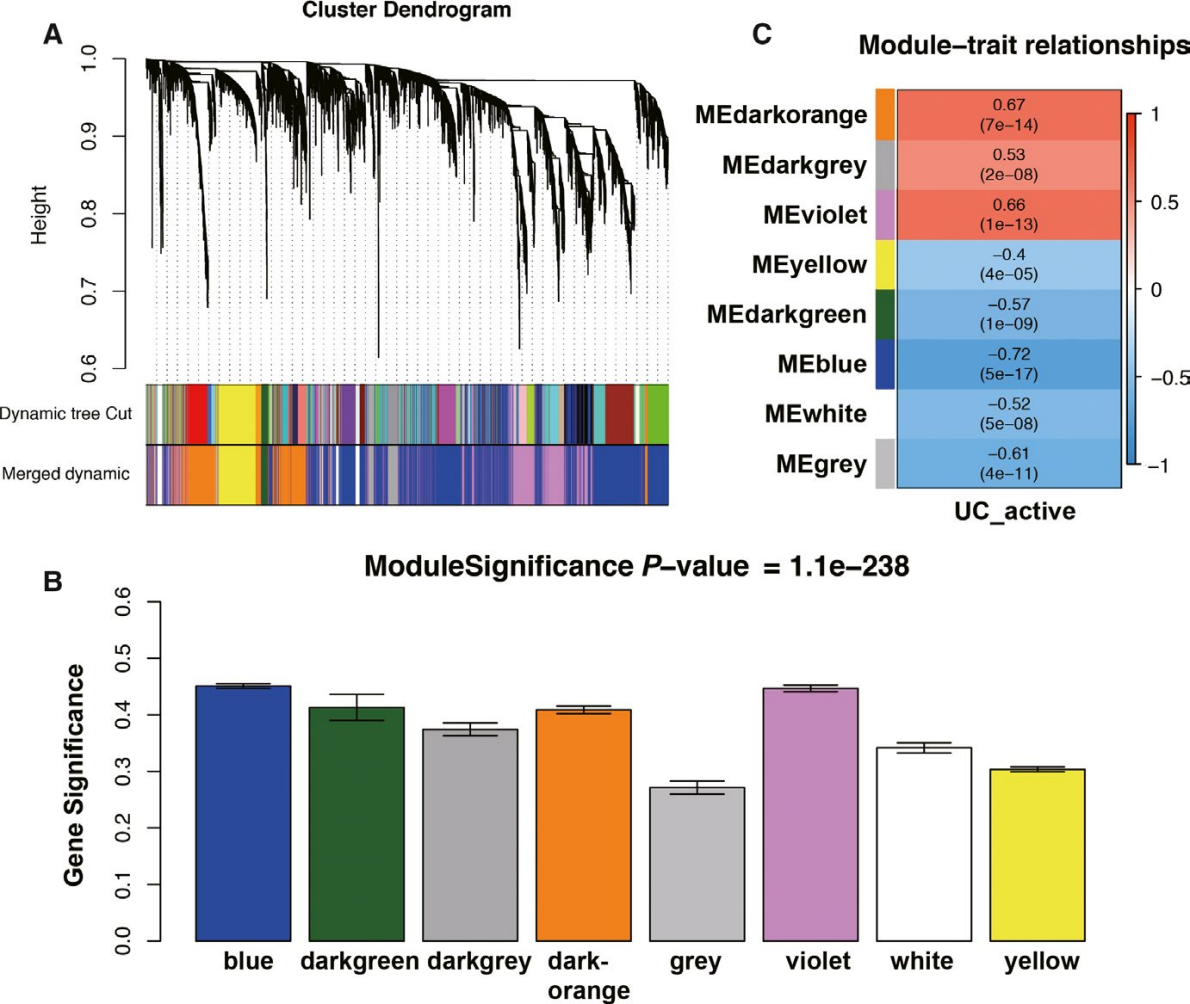
Identification of key modules related to active ulcerative colitis. A, Dendrogram of all differentially expressed genes clustered based on a dissimilarity measure (1‐TOM). B, Distribution of average gene significance and errors in the modules related to active ulcerative colitis. C, Heat map of the correlation between module eigengenes and active ulcerative colitis phenotype. TOM, topological overlap matrix

We used the ‘WGCNA’ R package to divide the DEGs into different modules by the average linkage clustering. First, a soft threshold *β* = 8 was calculated to construct a scale‐free network (Figure 2). Then, eight modules were identified, and two methods mentioned above were used to select modules highly associated with active UC (Figure 3A). The module significance (MS) of the blue module was higher than other modules (Figure 3B). In addition, the module eigengene (ME) showed the blue module was most relevant to the active UC (*r* = −.72, *P* = 5e‐17) (Figure 3C). Thus, our data indicated that the blue module was the key module associated with active UC. Meanwhile, all genes in the blue module were selected for the identification of hub gene.

### 3.3 Identification of hub gene

Both under the threshold of weighted and Pearson's correlation coefficients higher than 0.85 (cor.weighted > **|**0.85**|** and cor.standard > **|**0.85**|**), carboxypeptidase A6 (CPA6) with the highest weighted and Pearson's correlation coefficients was identified as the hub gene (Table 1).

**TABLE 1:** The hub genes in the blue module ranked by weight correlation and standard correlation

Genes	Cor.weighted	Cor.standard	FDR	LogFC
CPA6	−0.924722903	−0.883189089	5.33E‐30	−1.981883123
CAPN13	−0.929979708	−0.858434327	3.33E‐30	−2.354803505
CNNM2	−0.921634525	−0.858783941	4.92E‐30	−1.185747847
GLRA2	−0.913453025	−0.888426716	6.14E‐35	−2.446405842
CFB	0.877379415	0.85308747	1.92E‐29	1.952957759
KYNU	0.924612797	0.860956963	1.36E‐30	3.199680071

Abbreviations: FC, fold change; FDR, false discovery rate.

### 3.4 Validation of CPA6 expression

**FIGURE 4:**
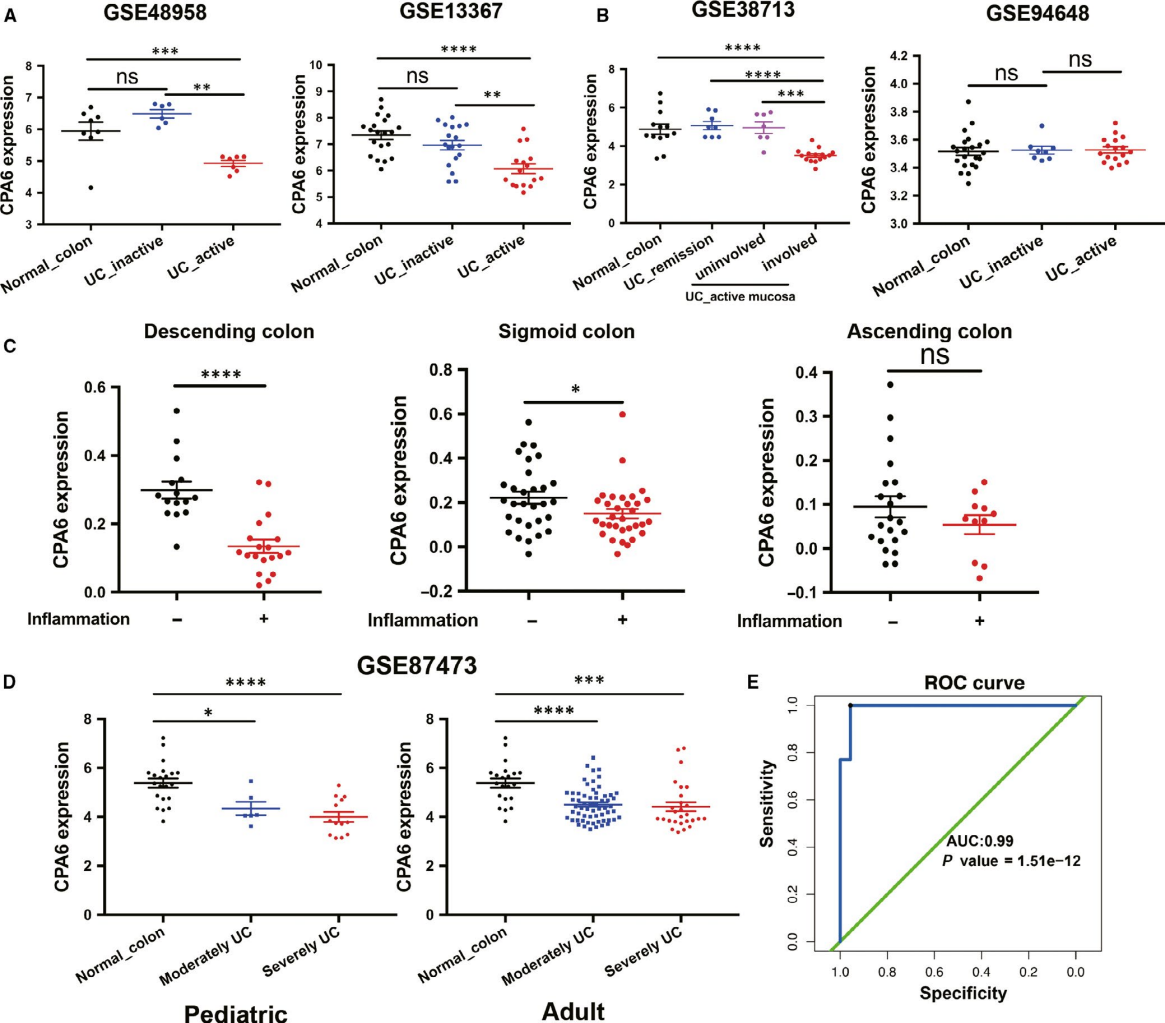
Validation of CPA6 expression. A, CPA6 expression in the active UC, inactive UC and normal colon according to the GSE48958 and GSE13367 data sets, respectively. B, CPA6 expression in the musoca of UC remission, uninvolved active UC, involved active UC and normal colon based on GSE38713 data set (left). CPA6 expression in the peripheral blood from the patients of active UC, inactive UC and normal control (right). C, According to the GSE11223 data set, the expression of CPA6 in the ascending colon, descending colon and sigmoid colon with and without inflammation. D, CPA6 expression in the moderately and severely UC samples from pediatric and adult samples. E, The ROC curve showed the diagnostic efficiency of CPA6 in GSE75214 data set. *P < .05, **P < .01, ***P < .001, ****P < .0001, ns, not statistically significant; ROC, receiver operating characteristic; UC, ulcerative colitis

In the test set of GSE48958 and GSE13367, although no significant differences were found between normal colon and inactive UC tissues, the expression of CPA6 was markedly down‐regulated in the active UC samples than that in inactive UC samples (Figure 4A). Moreover, we found that the CPA6 expression was specifically decreased in the involved mucosa of active UC, while no significant difference was found in remission UC and even the uninvolved mucosa from active UC (Figure 4B). There was no difference in peripheral blood (Figure 4B). In the UC patients of GSE11223 test set, compared with the mucosa without inflammation, we found the CPA6 expression was significantly reduced in the inflammatory mucosa in the descending colon, slightly decreased in the sigmoid colon, while no significant difference in the ascending colon (Figure 4C). However, no significant difference was found in the pediatric and adult patients with moderately and severely UC (Figure 4D). The ROC curve also indicated that CPA6 showed a great power in diagnosing active UC (AUC = 0.99, Figure 4E). These data suggested that CPA6 expression could be altered by inflammation, but there was no significant difference from the severity of inflammation.

### 3.5 Relationship between CPA6 expression and anti‐TNF treatment

**FIGURE 5:**
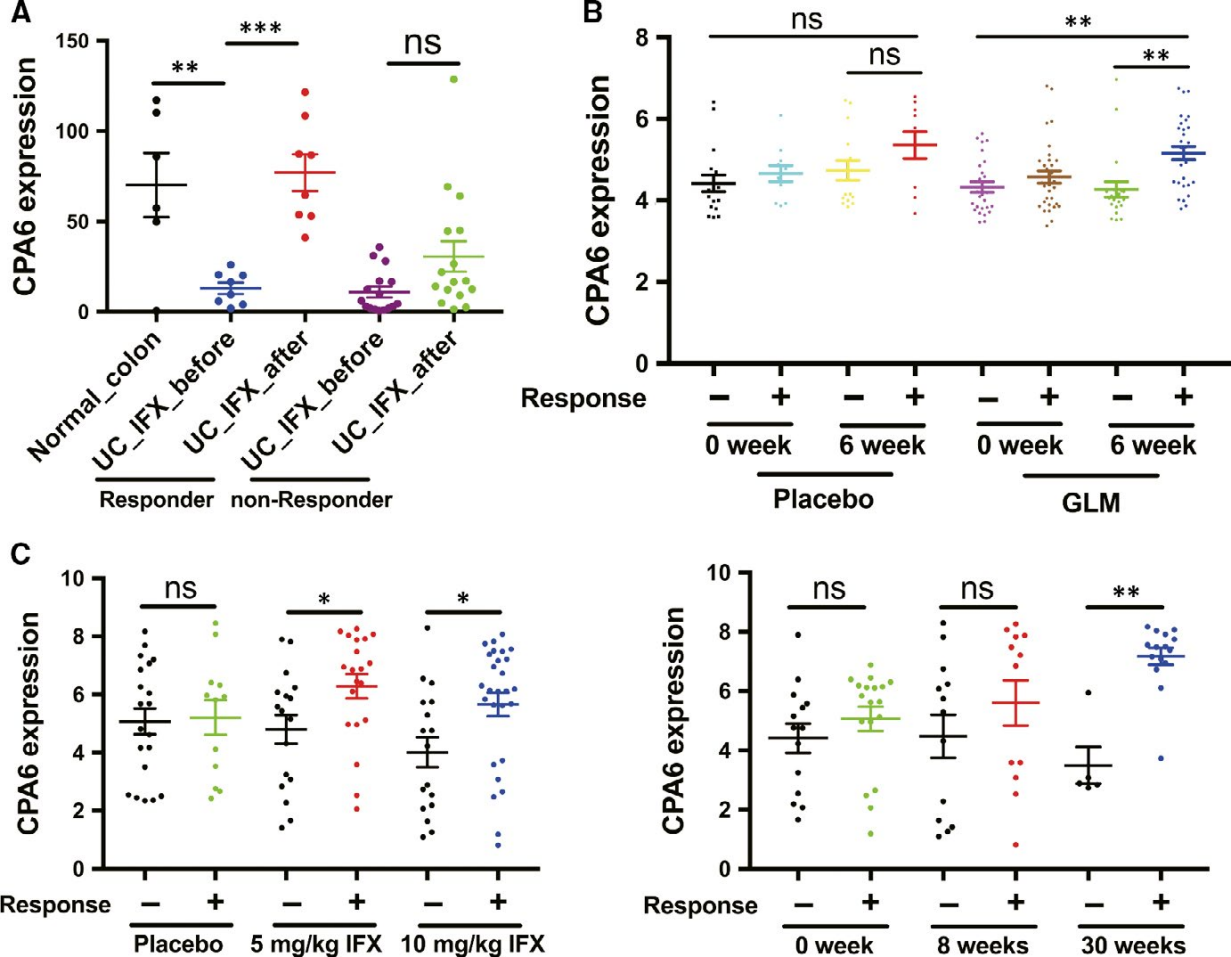
Relationship between CPA6 expression and anti‐TNF treatment. A, According to the GSE16879 data set, CPA6 expression was showed shown in UC patients IFX before and after IFX treatment. B, Based on the GSE92415 data set, UC patients were treated with GLM and placebo for 6 wk, respectively. The CPA6 expression was compared. C, Based on the GSE23597 data set, compare the effects of IFX doses (5 and 10 mg/kg, left) and the time of IFX administration (0, 8 and 30 wk, right) on CPA6 expression in patients with IFX response. *P < .05, **P < .01, ***P < .001 , GLM, golimumab; IFX, infliximab; ns, not statistically significant; TNF, tumour necrosis factor; UC, ulcerative colitis

Importantly, according to the GSE16879 data set, IFX treatment significantly increased CPA6 expression for UC patients who responded to IFX, while for UC patients who did not respond to IFX, CPA6 expression was not significantly changed after IFX treatment (Figure 5A). Similarly, GLM treatment for 6 weeks also increased CPA6 expression for UC patients who responded to GLM compared with the placebo (Figure 5B). We also found that the increase in CPA6 expression was not related to the dose of IFX, but to the time of administration based on the GSE23597 data set (Figure 5C).

### 3.6 Relationship between CPA6 expression and UC‐related colon cancer

**FIGURE 6:**
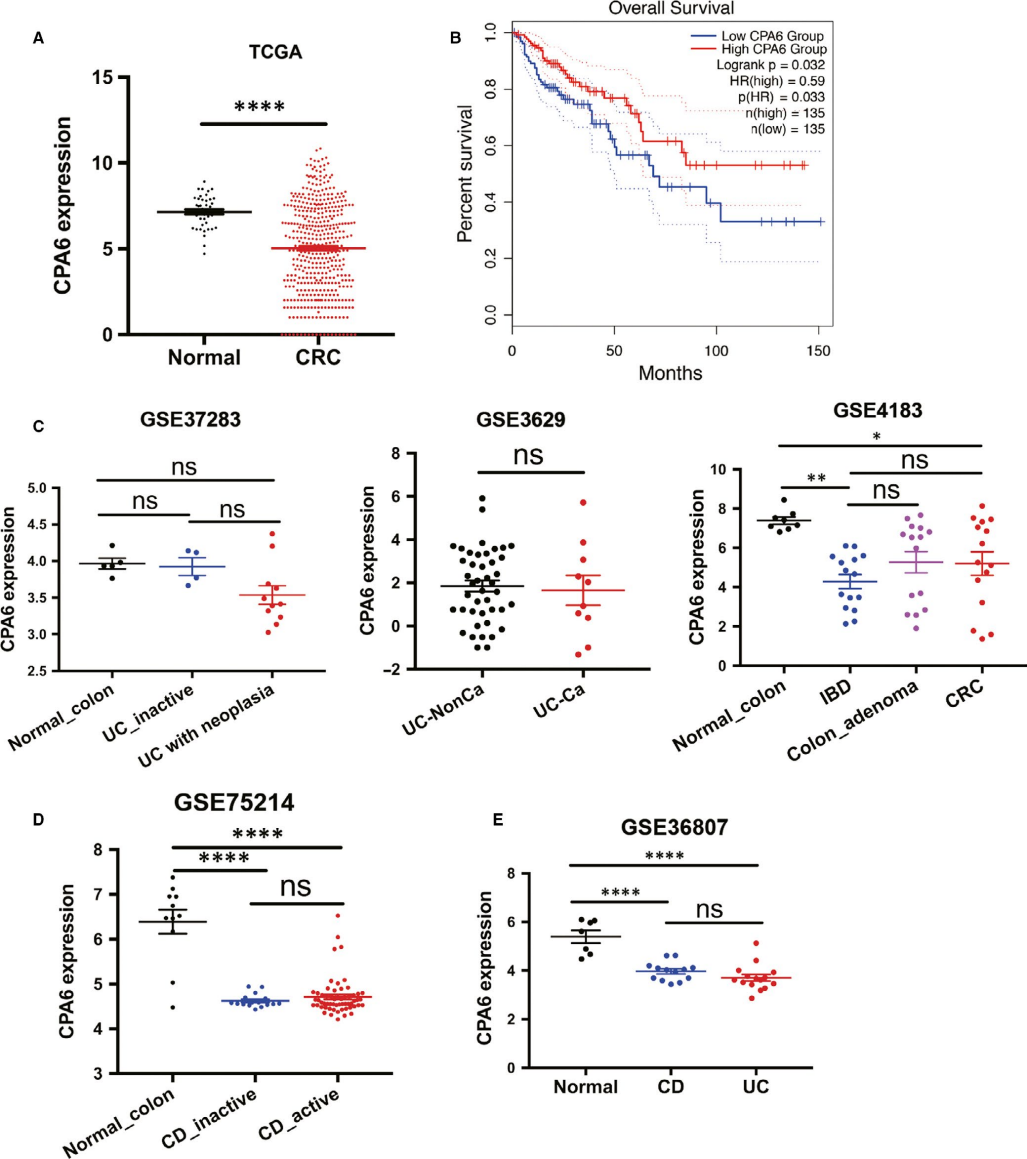
Relationship between CPA6 expression and UC‐related colon cancer. A, CPA6 expression was showed in the normal colon and CRC samples based on the TCGA database. B, The GEPIA2 database showed the effect of CPA6 expression on the OS of CRC. C, Comparison of CPA6 expression in normal tissues, UC/IBD without tumour (UC‐NonCa), UC‐related oncological lesions (UC‐Ca) and CRC. D, Comparison of CPA6 expression in the active CD, inactive CD and normal colon according to the GSE75214 data set. E, Comparison of CPA6 expression in normal tissues, UC and CD based on the GSE36807 data set. *P < .05, **P < .01, ****P < .0001, CD, Crohn's disease; CRC, colorectal cancer; IBD, Inflammatory bowel disease; ns, not statistically significant; OS, overall survival; UC, ulcerative colitis

Additionally, we further explored whether the lack of CPA6 expression is associated with UC carcinogenesis. Based on the TCGA and GEPIA2 databases, CPA6 expression was down‐regulated in CRC compared with normal tissues, and higher expression of CPA6 showed better overall survival (OS, *P* = .032), indicating that CPA6 tends to play a role in suppressing CRC (Figure 6A,B). However, CPA6 expression was not significantly different between UC/IBD and UC‐related neoplastic lesions (Figure 6C). In addition, we also compared the expression of CPA6 in CD. Although CPA6 expression in CD samples was also lower than that in the normal colon, it cannot distinguish between active CD and inactive CD based on the GSE75214 data set (Figure 6D). CPA6 expression levels were also close between UC and CD samples based on the GSE36807 data set (Figure 6E).

### 3.7 GSEA and GSVA

**FIGURE 7:**
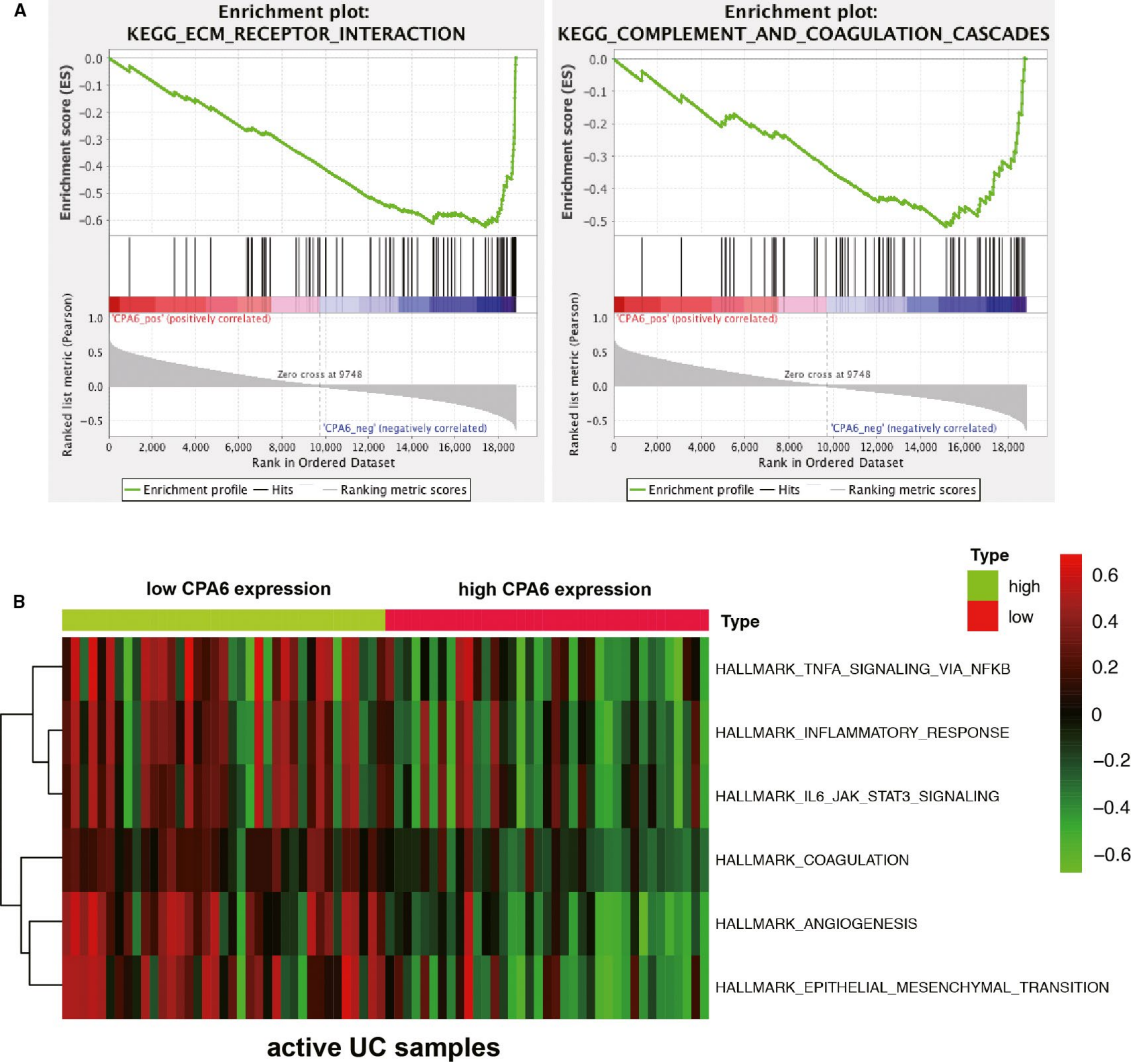
Gene set enrichment analysis (GSEA) and gene set variation analysis (GSVA). A, The GSEA found that ‘ECM receptor interaction’ and ‘complement and coagulation cascades’ gene sets were enriched in CPA6 low expression group based on the GSE75214 data set. B, Representative enriched gene sets via GSVA, such as ‘inflammatory response’, ‘TNFA signalling via NFKB’, ‘IL‐6/JAK/STAT3 signalling’ ‘angiogenesis’, ‘coagulation’ and ‘epithelial‐mesenchymal transition’ in active UC patients with low CPA6 expression. ECM, extracellular matrix; IL‐6, Interleukin‐6; NFKB, nuclear factor (NF)‐κB; TNFA, tumour necrosis factor α; UC, ulcerative colitis

To further explore underlying mechanism of CPA6 associated with KEGG pathways in the samples of active UC, GSEA was conducted and two gene sets of ‘ECM receptor interaction’ and ‘complement and coagulation cascades’ were significantly enriched (Figure 7A). Furthermore, the GSVA results demonstrated that inflammatory response, TNFA signalling via NFKB, IL‐6/JAK/STAT3 signalling, angiogenesis, coagulation and epithelial‐mesenchymal transition were highly enriched in active UC with low CPA6 expression (Figure 7B and Table 2).

**TABLE 2:** All the differentially enriched gene sets between low‐ and high‐CPA6 expression group in active UC patients

Gene set	Mean score difference	FDR
HALLMARK_ANGIOGENESIS	0.452757673	6.43E‐09
HALLMARK_COAGULATION	0.244467014	9.29E‐07
HALLMARK_EPITHELIAL_MESENCHYMAL_TRANSITION	0.412551039	2.48E‐06
HALLMARK_INFLAMMATORY_RESPONSE	0.302365549	9.88E‐04
HALLMARK_IL6_JAK_STAT3_SIGNALING	0.262078232	0.003591
HALLMARK_TNFA_SIGNALING_VIA_NFKB	0.296184822	0.003591

## 4 DISCUSSION

In this study, we firstly constructed a co‐expression network to identify key modules highly associated with active UC and the blue module was finally chosen as the key module. Furthermore, 6 genes were considered as hub genes because of both the cor.weighted > **|**0.85**|** and cor.standard > **|**0.85**|** (Table 1). Since CPA6 had both relatively higher cor.weighted (−0.9247) and cor.standard (−0.8832) among these hub genes, we eventually selected CPA6 for further validation.

Carboxypeptidase A6 (CPA6) was a member of the M14 metallocarboxypeptidase family and was discovered in the bioinformatics screening of the human genome in 2002.^34^, ^35^ Similar to other carboxypeptidase subtypes, in vitro experiment results showed that CPA6 cleaved the C‐terminal residue amino acid from several peptides, such as Met‐enkephalin‐Arg‐Phe, Big SAAS, or aldolase C terminus peptide.^34^, ^36^ It was reported that CPA6 mutations were closely related to the occurrence of Duane syndrome, a congenital eye movement disorder in which eye abduction is restricted or absent, and adduction is restricted.^37^ In addition, CPA6 mutations were also identified in patients with juvenile myoclonic and epilepsy.^38^, ^39^ These data suggested that CPA6 plays an important role in maintaining the normal physiological functions of the human body. Moreover, Bar et al^40^ found that CPE deficiency exacerbated experimental chronic colitis. However, the function of CPA6 in IBD is not yet understood (Figures 7).

In this study, we found that the expression of CPA6 was significantly decreased in active UC compared with inactive UC, and there was no significant difference between active CD and inactive CD (Figures 4A and 5D,E). Moreover, the decrease of CPA6 expression was only found in the descending colon mucosa affected by active UC, while no significant change was found in other mucosa sites and peripheral blood cells. The ROC curve showed a good power of CPA6 expression to diagnose active UC from inactive UC patients (Figure 4B‐D). Furthermore, we found that expression of CPA6 in IFX‐responsive UC patients increased after IFX treatment and was highly associated with the time of IFX administration, regardless of the IFX dose (Figure 4E,F). A review had demonstrated that metallocarboxypeptidase was an emerging drug target in biomedicine.^41^ More importantly, as reported by Lopes MW in 2016, knock‐down of CPA6 in zebrafish larvae reduced response to seizure‐inducing drugs.^42^ It was also reported that CPA6 gene mutations were also related to the response to metformin in patients with type 2 diabetes.^43^ Therefore, we can reasonably speculate that CPA6 was associated with anti‐TNF treatment combining the above results and literature analysis. In addition to being associated with to non‐tumour disorders, CPA6 expression had also been found to be dysregulated in several types of cancer, such as hepatocellular carcinoma and oral squamous cell carcinoma.^44^, ^45^ However, the role of CPA6 in CRC and even UC‐related tumours is unknown. Based on the TCGA database, we found that CPA6 acted as a tumour suppressive role and improved the OS of CRC patients (Figure 5A,B). However, CPA6 expression was not found to be different between UC and UC‐related tumour samples (Figure 5C). Thus, the effect of CPA6 on the development of UC‐related tumour requires further verification.

To further explore the potential mechanism of CPA6, we performed GSEA and GSVA analysis on the GSE75214 dataset, respectively. We found that extracellular matrix and coagulation were significantly enriched in UC patients with low CPA6 expression. Increasing evidence demonstrated the importance of extracellular matrix remodelling of the intestinal tissue in IBD and increased ECM deposition was found in UC patients.^46^, ^47^, ^48^ Our data also showed that the expression of some markers of ECM remodelling was up‐regulated in active UC (Table 3). Meanwhile, GSVA also indicated that the TNF‐α/NF‐κB pathway, the inflammatory response and IL6/JAK/STAT3 pathway were significantly activated in patients with lower expression of CPA6. This partially explained the alteration of CPA6 expression after IFX and GLM treatment, because it may be involved in regulating the immune microenvironment of UC. Collectively, these data indicated that CPA6 expression was usually down‐regulated in active UC, probably through regulating ECM and immune response. However, the function and mechanism of CPA6 requires further investigation.

**TABLE 3:** Comparison the expression of genes associated with ECM remodelling in active UC than inactive UC through ‘limma’ analysis

Genes	LogFC	FDR
MMP1	3.752338554	2.72E‐16
MMP10	2.286023546	6.45E‐16
MMP12	2.888798576	3.90E‐17
MMP13	0.646429326	0.019461511
MMP14	0.6858979	5.15E‐08
MMP19	0.772283658	8.04E‐07
MMP2	1.367144713	8.45E‐07
MMP25	0.52758486	4.20E‐05
MMP3	4.319764475	1.88E‐18
MMP7	3.409456808	2.55E‐18
MMP9	2.050124066	3.03E‐13
VIM	0.973434638	1.18E‐08
ECM1	0.570814803	6.54E‐09
TWIST1	0.411068937	4.98E‐05
ZEB1	0.624334045	3.72E‐05
ITGA2	1.320094815	2.44E‐11
ITGA4	0.89648597	8.88E‐08
ITGA5	1.267420214	2.86E‐07
ITGA8	0.952246372	1.46E‐08
ITGAL	0.778865689	1.46E‐06
ITGAM	0.778755105	8.75E‐06
ITGAV	0.761334305	5.73E‐15
ITGAX	1.781822677	2.42E‐14
ITGB2	1.150028477	2.10E‐10
ITGB3	0.769789545	3.34E‐07

Abbreviations: ECM, extracellular matrix; FC, fold change; FDR, false discovery rate; UC, ulcerative colitis.

## 5 CONCLUSION

In summary, to identify and verify hub genes highly associated with active UC, we used WGCNA algorithm to construct a co‐expression network and indicated the down‐regulation of CPA6 was highly related to active UC, probably through regulating ECM and immune responses. Furthermore, anti‐TNF treatment could rescue the CPA6 expression. Thus, our data have important clinical significances and will be helpful in the diagnosis of active UC.

## CONFLICT OF INTEREST

The authors confirm that there are no conflicts of interest.

## AUTHOR CONTRIBUTIONS

**Haizhou Wang:** Conceptualization (lead); Data curation (lead); Investigation (lead). **Meng Zhang:** Data curation (equal); Investigation (equal). **Mengna Zhang:** Data curation (equal); Methodology (equal). **Fan Wang:** Supervision (equal); Validation (equal); Writing‐review & editing (equal). **Jing Liu:** Funding acquisition (equal); Supervision (equal); Writing‐review & editing (equal). **Qiu Zhao:** Conceptualization (lead); Funding acquisition (lead); Supervision (lead).

## Supporting information

Figure S1

## Data Availability

The data sets used and/or analysed during the current study are available from the corresponding author on reasonable request. The public data source: TCGA database (https://portal.gdc.cancer.gov/), GEO database (https://www.ncbi.nlm.nih.gov/geo/) and GEPIA2 database (http://gepia2.cancer-pku.cn).
