# Supplementary material for: Carboxypeptidase A6 was identified and validated as a novel potential biomarker for predicting the occurrence of active ulcerative colitis
Source: J Cell Mol Med. 2020 Jun 22;24(15):8803–13. doi: 10.1111/jcmm.15517 (PMC7412415; doi:10.1111/jcmm.15517)
Supplement: Supplementary file 1 — Figure S1 [file JCMM-24-8803-s001.zip › jcmm15517-sup-0002-legend.docx]

**Figure S1:** Samples clustering to detect outliers in the GSE75214 data set.
